# Special Issue—Diabetes Mellitus: Current Research and Future Perspectives, 2nd Edition

**DOI:** 10.3390/jpm16060325

**Published:** 2026-06-17

**Authors:** Evelina Maines, Roberto Franceschi

**Affiliations:** Pediatric Diabetology Unit, S. Chiara Hospital of Trento, Largo Medaglie d’Oro, 9, 38122 Trento, Italy; roberto.franceschi@asuit.tn.it

In 1922, Leonard Thompson, a 14-year-old patient with severe Type 1 Diabetes (T1D), became the first patient to receive an insulin injection. By 1923, a pharmaceutical laboratory in Germany began mass-producing insulin, paving the way for a new era [1]. Over the next century, the insulin molecule underwent several modifications to enhance its stability and duration of action. This evolution was accompanied by advancements in insulin delivery systems, the accuracy of glucose measurement, and the development of tools for lifestyle and dietary management. Today, the transition toward precision medicine is shifting the paradigm from reactive glucose management to proactive, individualized, and prospective care.

Similarly, the management of Type 2 Diabetes (T2D) has undergone a major transformation, including greater attention to preventive care and the introduction of innovative drug classes, such as sodium–glucose cotransporter 2 (SGLT-2) inhibitors and glucagon-like peptide-1 receptor agonists (GLP-1RAs).

The Special Issue “Diabetes Mellitus: Current Research and Future Perspectives” provides a comprehensive overview of how precision medicine empowers clinicians to enhance diabetes care across several critical domains, such as earlier detection and staging, secondary and long-term preventive strategies, and advanced technology integration.

Earlier Detection and Staging: Diabetic ketoacidosis (DKA) still occurs in 15% to 70% of children at T1D onset, leading to considerable morbidity, hospitalization rates, and even mortality [2]. Fanti et al. investigated factors associated with DKA to reduce its incidence and severity, as well as to optimize preventive resource allocation [Contribution Fanti et al.]. The authors observed a high frequency of DKA among children aged 10–14 years, consistent with previous reports. The impact of diabetes awareness campaigns on the frequency of DKA at diagnosis varies significantly. To be effective, these campaigns must adhere to specific rules regarding target population, modality, and minimal duration.

Antibody screening of the general population is a promising T1D preventive strategy. Studies involving individuals at risk for T1D have demonstrated that the disease is a continuum progressing through distinct, identifiable stages before symptomatic onset, offering a critical window for early identification. Stage 1 is defined by the presence of β-cell autoimmunity (two or more islet autoantibodies, IAb+) with normoglycemia and is pre-symptomatic; Stage 2 involves β-cell autoimmunity with dysglycemia and remains pre-symptomatic; Stage 3 marks the onset of symptomatic disease [3]. Interestingly, Mănescu et al. investigated what happens before stage 1 (Stage 0) in individuals who will eventually develop T1D [Contribution Mănescu et al.]. By synthesizing influential research from the fields of epigenetics, omics, and microbiota, they identified candidate biomarkers that can predict T1D before the onset of islet autoimmunity, suggesting that the prophylactic window may begin at birth. Although these biomarkers seem promising, they still require clinical validation.

Screening children in preclinical stages for IAb+ may help identify at-risk individuals, allowing for systematic follow-up and the potential prevention of DKA. An early diagnosis at Stage 1 or 2, or potentially at Stage 0, provides the opportunity to educate families on the signs of Stage 3 and enables participation in clinical trials aimed at delaying disease progression [4]. As the therapeutic landscape evolves from disease treatment to disease delay, and ideally toward disease prevention, it is crucial to identify and validate “Stage 0” biomarkers. In the era of precision medicine, these new data will enable early intervention with the potential to delay, modify, or prevent autoimmunity and consequently T1D in at-risk children.

In a global first, the Italian Parliament approved a law in September 2023, mandating nationwide T1D screening for the pediatric population (1–17 years) [5]. In the near future, artificial intelligence (AI) could support the development of predictive models based on Stage 0 biomarkers to estimate diabetes risk across diverse groups and refine screening selection.

Beyond preventing DKA, early diagnosis may contribute to preserving endogenous insulin secretion in the majority of children leading to more favorable clinical presentation and reducing insulin needs [6].

Similarly, T2D onset manifests across various stages and degrees of severity, resulting in distinct clinical phenotypes. The known risk factors for T2D encompass genetic, metabolic, and environmental interactions that drive disease onset. The individual predisposition stems from non-modifiable contributors, such as ethnicity and family history, alongside modifiable risks like obesity, low physical activity, and an unhealthy diet. All these contributors trigger the core pathophysiological drivers of T2D, including beta-cell dysfunction, insulin resistance, the role of visceral adipose tissue, and chronic systemic inflammation. To address this complexity, Pfützner et al. developed a method for individual phenotyping using functional biomarkers [Contribution Pfützner et al.]. This approach may facilitate a more pathophysiology-oriented and personalized diagnostic strategy, crucial for preventing, stabilizing, and potentially reversing T2D.

Secondary and long-term preventive strategies: Disease-modifying therapies are being developed to delay progression to Stage 3 T1D in people with IAb+. In 2022, the US Food and Drug Administration (FDA) approved teplizumab, the first treatment to delay the T1D Stage 3 onset. Other immunomodulatory and/or immunosuppressive agents to delay the progression of beta-cell destruction are under approval. Teplizumab is a humanized monoclonal antibody which binds with high affinity to CD3 chains, preventing the activation of T cells, in particular CD8+, which are involved in the destruction of beta-cells [7]. The TN-10 study (NCT01030861), which enrolled relatives of patients with T1D at high risk of developing T1D, demonstrated that a two-week treatment with Teplizumab delayed Stage 3 onset by a median of 2.7 years [8,9]. Notably, the response to teplizumab was more prominent in relatives with lower C-peptide levels, suggesting that teplizumab may be more beneficial for individuals with a more significant loss of beta-cell function [8]. Additional potential response indicators currently being explored include baseline variations in T cell subsets [7,10]. Recently, significant differences in the baseline levels of 15 different metabolites were identified between responders and non-responders. Specifically, responders exhibited higher baseline levels of tricarboxylic acid (TCA) cycle intermediates, amino acid derivatives, and microbial metabolites. This metabolomic profile suggests that Teplizumab responders possess enhanced mitochondrial activity, and greater immune tolerance at the start of treatment. These findings may suggest that baseline metabolite profiles could serve as predictive biomarkers for treatment response [11].

Also, in T2D, pre-diabetes represents a critical window for preventive care, as it precedes clinical disease and contributes to long-term adverse outcomes. Conventional T2D therapy primarily targets metabolic indices such as HbA1c levels using standardized approaches. This treatment model does not always consider individual phenotypes and care setting. As the pathophysiology and underlying mechanisms of T2D become increasingly understood, precision medicine should be implemented to tailor treatments effectively. This could be achieved through molecular genetic tools and specific biomarkers to define disease at onset, progression and ultimately therapeutic response. Advancing toward highly personalized and automated therapeutic strategies, the “digital twin”, a virtual representation of an individual patient, could revolutionize diabetes management by integrating data to predict physiological responses, moving from traditional care to a truly personalized approach. However, several critical challenges must be addressed before these findings can be translated into routine practice; specifically, questions remain regarding their robust clinical validation in diverse cohorts, the practical feasibility of large-scale implementation in real-world healthcare settings, and the broader generalizability of the results across heterogeneous populations.

Advanced Technology Integration: Over the past decade, rapid technological advances have led to the introduction of sophisticated Continuous Glucose Monitoring (CGM) systems. These systems not only provide near-real-time glucose readings, but also communicate with state-of-the-art insulin pumps to automate insulin delivery based on glucose trends. Automatic Insulin Delivery (AID) systems—consisting of an insulin pump, a CGM sensor, and an algorithm that determines insulin dosing—regulate administration in response to sensor-detected data [12]. The use of AID systems is increasing across all age groups with T1D [13], and these systems are effective in improving metabolic control and Patient-Related Outcomes (PROs) [14,15,16]. A recent meta-analysis of 11 trials assessing the efficacy of AID systems compared with other insulin regimens reported that time in range (TIR) increased by an average of 11.5%, and at the same time glycated hemoglobin (HbA1c) levels were reduced by an average of 0.41% for use greater than 6 months [14]. These findings are supported by real-world data confirming that AID systems are more effective in achieving glycemic goals than conventional therapies [15].

Hybrid closed-loop (HCL) systems partially automate insulin dosing, requiring only manual boluses at mealtime or for occasional correction [16]. While these systems have already driven significant improvements in glucose control and automation, the ultimate goal in the field is the development of long-term, implantable glucose sensors and fully AID systems [17]. In the near future, such systems could leverage AI to maintain glucose levels within the physiological range, eliminating the need for human intervention. Ensuring clinical safety and data security is paramount for the successful implementation of AI and data-driven tools in diabetes management. However, integrating these tools into existing clinical practice remains a challenge, due to practically, ethically, and logistical hurdles. It also demands a transformation in healthcare infrastructures to ensure these innovations are accessible to all patients. All stakeholders involved in diabetes care must invest in research to harness the benefits of AI while mitigating the risks of an increasingly digitalized healthcare system.

**Conclusions**

The Special Issue “Diabetes Mellitus: Current Research and Future Perspectives” covers a broad spectrum of current research and emerging outlooks in the field of diabetes.

Through autoimmunity screening, new biomarkers and genetic risk scores, we can now identify individuals in the “presymptomatic” stages of the disease [Contribution Mănescu et al.]. This advances a critical window for disease-modifying therapies aimed at delaying disease progression and for implementing targeted strategies to prevent DKA [Contribution Minerba et al.] [Contribution Fanti et al.].

The core of precision medicine in diabetes is the recognition of T1D or T2D “subtypes”, each of which has distinct features that might be amenable to specific interventions. By decoding this disease heterogeneity [Contribution Pfützner et al.], we can match the right therapy to the right patient based on their specific immune or metabolic profile [Contribution Jantz et al.] or specific care setting [Contribution Sinclair et al.]. For instance, Maltoni et al. observed that hypertriglyceridemia at T1D onset is independently associated with higher insulin resistance (IR), suggesting that this readily available marker can anticipate individual insulin requirements at T1D diagnosis [Contribution Maltoni et al.]. Yoon et al. investigated sex- and age-specific cutoff values for Homeostatic Model Assessment of Insulin Resistance (HOMA-IR) for improving personalized risk assessment for dysglycemia in T2D patients [Contribution Yoon et al.].

Today, artificial intelligence is poised to become the new protagonist, evolving from a simple tool into the driving force of future diabetes management, as highlighted by the “digital twin” representation [Contribution Cáceres-Gutiérrez et al.].

The integration of precision medicine represents a promising avenue for improving long-term outcomes. Popa et al. documented that caffeine intake, glucose, and cholesterol are interconnected and proportionally related, suggesting the necessity of multifaceted preventive strategies [Contribution Popa et al.]. Gisinger et al. stressed the need for targeted immune preventive strategies in T2D patients with specific comorbidity patterns [Contribution Gisinger et al.]. Roshan et al. highlighted that insulin resistance and markers of liver steatosis and fibrosis may be associated with restrictive lung impairment, emphasizing the need for multidisciplinary follow-up [Contribution Roshan et al.].

In conclusion, the management of diabetes is undergoing a paradigm shift, moving away from standardized protocols toward highly personalized therapeutic strategies.

## Data Availability

Not applicable.
